# Proximate mechanisms affecting seasonal differences in migration speed of avian species

**DOI:** 10.1038/s41598-018-22421-7

**Published:** 2018-03-07

**Authors:** Heiko Schmaljohann

**Affiliations:** 1 Institute of Avian Research “Vogelwarte Helgoland”, An der Vogelwarte 21, 26386 Wilhelmshaven, Germany; 2 Institute for Biology und Environmental Sciences (IBU), Carl von Ossietzky University of Oldenburg, Carl-von-Ossietzky-Straße 9-11, D-26129 Oldenburg, Germany

## Abstract

Faster migration in spring than in autumn seems to be a common pattern in birds. This has been ultimately explained by seasonally different selection pressures. Variation in migration speed is proximately caused by adjusting travel speed (distance covered during flight) and/or stopover duration (times when birds rest and refuel). Yet, it remains unclear whether individual seasonal differences in migration speed match the common pattern and what the precise role of the proximate, behavioural mechanisms for adjusting migration speed is. By reviewing 64 studies of 401 tracks, I show that in waders, gulls, swifts, and songbirds speeds were significantly higher in spring, while the opposite was the case in waterfowl and owls. Thus, the ultimate mechanisms selecting for a faster migration in spring might not consistently act across bird groups. Breeding latitude, migration strategy, migration distance, flight style, body mass, and sex did not explain seasonal differences in speed. The ratio between spring and autumn total stopover duration of 257 bird tracks significantly negatively affected the seasonal migration speed ratio of the same individuals in a comparative analysis accounting for shared ancestry. Seasonal variation in stopover duration appears thus to be the main biological mechanism regulating seasonal differences in migration speed.

## Introduction

The general phenomenon that migratory birds travel faster in spring than in autumn has been ultimately explained by seasonally different selection pressures^1^. It is generally accepted that selection favours a time minimizing migration strategy by maximizing speed of migration in spring. This strategy would thus maximize individual fitness as birds arrive early at their breeding areas^2–5^. A slower and likely less energetically costly migration strategy is believed to be favoured in autumn^6,7^. Yet, there are counter-examples to this general phenomenon in some major groups of bird migrants, i.e., geese^8^, ducks^9^, swans^10^, storks^11^, raptors^12^, waders^13^, gulls^14^, and songbirds^15^. These might suggest that there is some advantage for individuals of these species to have a higher total speed of migration, i.e., total migration distance (km) divided by total duration of migration (days), in autumn and a less costly migration in spring^8^. Thus, the ratio between total speed of migration in both seasons might be indicative for the experienced selection pressures favouring either to maximize speed of migration or to minimize energy costs of migration. Nilsson *et al*.^1^ highlighted in their review that it will be an important future task to perform detailed comparisons between different bird groups to assess potential differences in the selection pressures likely shaping their seasonal-specific migration strategy and thus migration speed.

While ultimate mechanisms explain “*why*” a certain trait has evolved, proximate, behavioural mechanisms explain “*how*” it is realized. A bird migrant could achieve a higher total speed of migration in one season by increasing the average distance covered non-stop per day or night (travel speed)^16^ and/or decreasing the time spent not travelling, i.e., reducing stopover duration^17^. The former excludes any time spent on the ground and depends on airspeed^18,19^, wind availability^20^, wind selectivity^21,22^, atmospheric conditions^23,24^, and the time spent flying per migratory stage^25^. During stopover, migrants rest on the ground and may fuel energy stores for the upcoming migratory flight, which is influenced among others by bird’s physiological state, food availability, day length, temperature, and predation risk^26,27^. Seasonal differences in any of these traits affecting travel speed and/or total stopover duration might therefore cause total speed of migration to be season specific^27^. In the majority of the tracking studies considered by Nilsson *et al*.^1^, the seasonal differences in travel speed were much smaller than those of stopover duration suggesting that the latter could explained most of the seasonal variation in total speed of migration. This accounts for the fact that the rate of accumulating energy is much slower than the rate of spending energy at flight^28^. Birds therefore spend generally more time on the ground than travelling during migration^29–31^. Yet, assessing whether seasonal variation in either proximate, behavioural mechanism significantly explains seasonal variation in total speed of migration still remains a major challenge in movement ecology.

The first objective of this study was to assess whether seasonal differences in total speed of migration were generally consistent across different bird groups. I reviewed the recent literature on the topic and analysed potential seasonal differences in total speed of migration at the individual-level (rather than population level^1^) by comparing a bird’s spring estimate with its autumn estimate for different bird groups (Table 1). These included waterfowl (Anseriformes), tubenoses (Procellariiformes), bustards (Otidiformes), storks (Ciconiiformes), raptors (Accipitriformes), waders (Charadriiformes: Charadrii), gulls (Charadriiformes: Lari), owls (Strigiformes), swifts (Apodiformes), cuckoos (Cuculiformes), rollers (Coraciiformes), hoopoes (Bucerotiformes), and songbirds (Passeriformes). The second objective was to assess whether ecological (such as breeding latitude, bird’s flight style, migration strategy, migration distance, sex) and morphological traits (such as body mass) may affect seasonal differences in total speed of migration, computed as the ratio between the corresponding individual values for spring and autumn (*Q*_*speed of migration*_)^1^, by controlling for phylogeny. The third objective was to assess the effect of seasonal differences in total stopover duration, as the ratio between the corresponding individual values for spring and autumn (*Q*_*stopover*_), on *Q*_*speed of migration*_, while accounting for shared ancestry among species^32^.

**Table 1:** Seasonal differences in total speed of migration for different bird groups. Number of studies and sample size of individual tracks for which total speed of migration was available for both seasons. The seasonal median and first and third quartiles of bird group’s total speed of migration are shown. For individuals being sexed in the original studies, sex-specific medians and first and third quartiles are also presented. *Q*_*speed of migration*_ is calculated as the decimal logarithm of the ratio between spring and autumn total speed of migration.

Bird group	Number of studies	Number of individuals	Total speed of migration in spring Median [25^th^, 75^th^ quantile] (km/day)	Total speed of migration in autumn Median [25^th^, 75^th^ quantile] (km/day)	Ratio between spring and autumn total speed of migration	*Q*_*speed of migration*_
Anseriformes (waterfowl)	3	22	52 [43, 64]	81 [67, 96]	0.64	−0.19
Males	2	17	52 [41, 64]	84 [78, 93]	0.67	−0.17
Females	2	4	48 [44, 67]	77 [62, 92]	0.57	−0.24
Procellariiformes (tubenoses)	1	10	668 [569, 743]	779 [669,868]	0.86	−0.07
Males	1	3	726 [712, 737]	881 [835, 905]	0.82	−0.09
Females	1	7	595 [550, 694]	686 [649, 803]	0.87	−0.06
Otidiformes bustards (only males)	1	4	94 [90, 106]	172 [166, 212]	0.55	−0.26
Ciconiiformes storks (sex unknown)	2	10	155 [113, 196]	193 [159, 267]	0.79	−0.10
Accipitriformes raptors	11	66	184 [137, 236]	154 [118, 210]	1.20	0.08
Males	5	16	213 [162, 272]	184 [151, 246]	1.16	0.06
Females	9	42	182 [140, 218]	147 [113, 187]	1.24	0.09
Charadriiformes waders	11	47	253 [174, 338]	185 [148, 210]	1.37	0.14
Males	9	29	282 [198, 341]	197 [160, 214]	1.43	0.16
Females	4	8	271 [216, 340]	180 [149, 376]	1.51	0.18
Charadriiformes gulls	6	41	134 [88, 211]	54 [23, 237]	2.50	0.40
Males	3	12	106 [77, 198]	60 [34, 165]	1.78	0.25
Females	3	4	133 [114, 215]	47 [19, 103]	2.83	0.45
Strigiformes owls (only males)	1	5	59 [51, 63]	165 [90, 220]	0.36	−0.44
Apodiformes swifts	2	17	363 [312, 600]	236 [170, 301]	1.54	0.19
Males	1	11	555 [338, 617]	275 [200, 330]	2.02	0.31
Cuculiformes cuckoos	1	3	85 [80, 104]	62 [60, 66]	1.37	0.14
Males	1	2	104 [94, 113]	60 [60, 61]	1.71	0.23
Females	1	1	74	71	1.04	0.02
Coraciiformes “rollers”	1	4	152 [146, 175]	102 [93, 112]	1.49	0.17
Males	1	2	193 [175, 211]	102 [99, 106]	1.88	0.27
Females	1	2	143 [140, 146]	101 [94, 108]	1.42	0.15
Bucerotiformes “hoopoes” (only females)	1	2	142 [132, 153]	100 [90, 109]	1.43	0.16
Passeriformes songbirds	25	170	154 [111, 211]	90 [71, 130]	1.71	0.23
Males	20	103	168 [112, 222]	98 [72, 130]	1.71	0.23
Females	11	35	174 [111, 242]	90 [74, 120]	1.93	0.29

## Results

### Seasonal differences in total speed of migration

**Figure 1:**
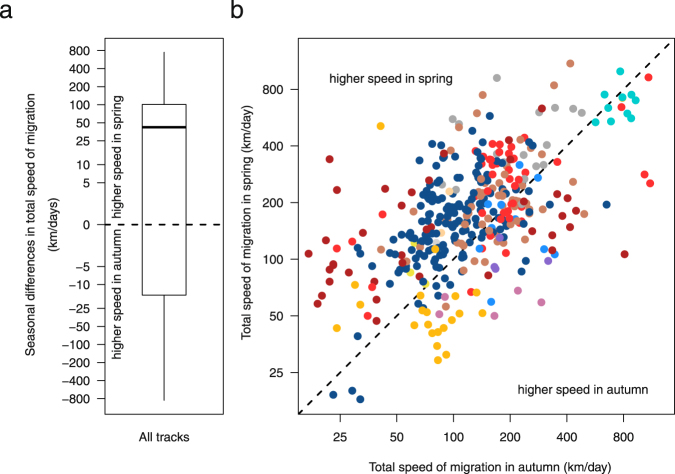
Individual seasonal differences (n = 401) in total speed of migration. (**a**) Individual seasonal differences in total speed of migration for all birds of all bird groups. The box plot shows the median and 25^th^ and 75^th^ percentiles; the whiskers indicate the values within 1.5 times the interquartile range. Dashed line indicates no seasonal differences in total speed of migration. (**b**) Spring individual total speed of migration plotted against the corresponding individual autumn value. Colours indicate different bird groups (waterfowl = orange, shearwater = cyan, bustards = purple, storks = light blue, raptors = light brown, waders = light red, gulls = dark red, owls = light purple, swifts = grey, cuckoos = yellow, rollers = light beige, hoopoes = light grey, and songbirds = dark blue). Dots above the dashed line represent individuals with higher total speed of migration in spring than in autumn. All axes are on a logarithmic scale.

**Figure 2:**
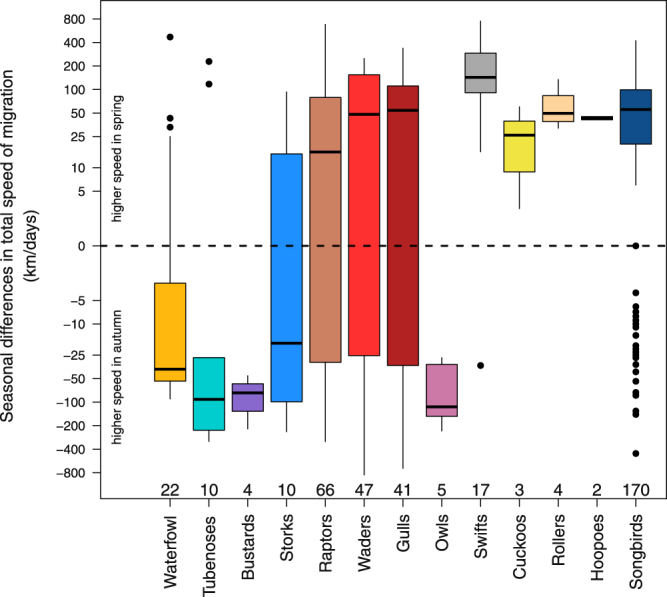
Individual seasonal differences in total speed of migration for different bird groups. Seasonal differences in total speed of migration as the individual spring value minus its corresponding autumn value for each bird group. Positive values indicated a higher total speed of migration in spring than in autumn and negative values the opposite. The box plots show the median and 25^th^ and 75^th^ percentiles; the whiskers indicate the values within 1.5 times the interquartile range; outliers are indicated by black dots. Different colours indicate different bird groups (waterfowl = orange, shearwater = cyan, bustards = purple, storks = light blue, raptors = light brown, waders = light red, gulls = dark red, owls = light purple, swifts = grey, cuckoos = yellow, rollers = light beige, hoopoes = light grey, and songbirds = dark blue). Sample sizes, i.e., number of individuals, are given above the corresponding bird groups. Y-axis is on a logarithmic scale.

**Figure 3:**
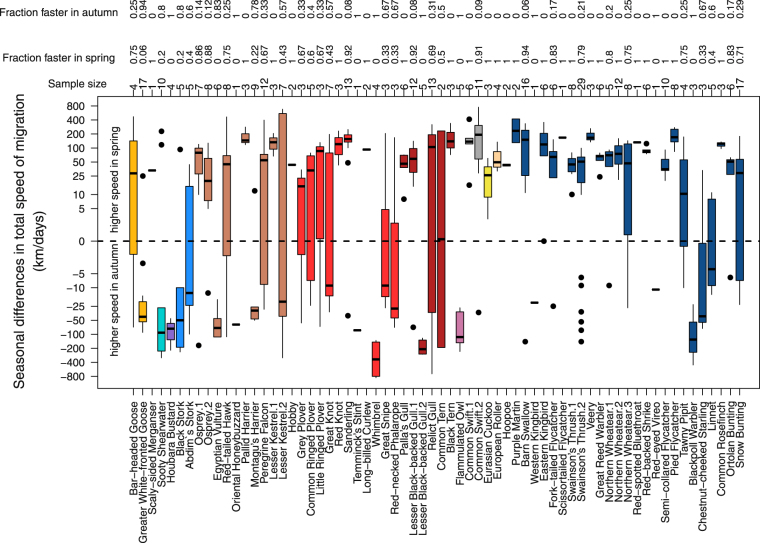
Individual seasonal differences in total speed of migration per species/population. Seasonal differences in total speed of migration as the individual spring value minus its corresponding autumn value for each species/population. Positive values indicated a higher total speed of migration in spring than in autumn and negative values the opposite. The box plots show the median and 25^th^ and 75^th^ percentiles; the whiskers indicate the values within 1.5 times the interquartile range; outliers are indicated by black dots. Numbers of individual tracks per species/population are given next to the plot and the fractions of individual tracks with faster spring/autumn migration compared to autumn/spring migration are given above the corresponding sample size. Different colours indicate different bird groups (waterfowl = orange, shearwater = cyan, bustards = purple, storks = light blue, raptors = light brown, waders = light red, gulls = dark red, owls = light purple, swifts = grey, cuckoos = yellow, rollers = light beige, hoopoes = light grey, and songbirds = dark blue). Different populations/studies of the same species were distinguished by different numbers, cf. Table S1. Y-axis is on a logarithmic scale.

The majority of individual bird tracks (276 out of 401, 69%) showed a higher total speed of migration in spring, whereas 124 of these (31%) migrated faster in autumn (Fig. 1). One individual (0.2%) had equal speeds in both seasons. In raptors, waders, gulls, swifts, cuckoos, rollers, hoopoes, and songbirds, more than half of all individuals showed higher total speed of migration in spring (Table 1, Fig. 2), whereas the opposite pattern was observed in waterfowl, tubenoses, bustards, storks, and owls (Table 1, Fig. 2). Among the groups showing significant seasonal differences (Table 1), the strongest effect was found in gulls, songbirds, and swifts, which migrating on average about 250%, 171%, and 154% faster in spring, respectively, while owls migrated about 250% and waterfowl about 156% faster in autumn (Table 1). At the level of the tracked species/population, the median of the individual seasonal differences in total speed of migration, with positive values indicating higher speeds in spring, was above zero in 45 out of the 66 cases, below zero in 21 (Fig. 3). The fraction of individuals migrating faster in either season varied between the species/population and also within bird groups (Fig. 3). For each species/population, I ran a one-sided Wilcoxon signed rank test to assess whether total speed of migration was higher in spring than in autumn (Table S1). Considering these results in a meta-analysis revealed that overall birds migrated significantly faster in spring than in autumn (Z-weighted method: z = 8.1, P < 0.0001, n_species/population_ = 57, cf. Table S1).

There were 22 species/populations tracking at least one individual per sex (Table 1). In four species, sample size of both sexes was sufficiently large to assess for sex-specific seasonal differences in total speed of migration, but there was no significant difference (Wilcoxon rank sum tests: Osprey *Pandion haliaetus*, n_males_ = 5, n_females_ = 10, W = 34, p = 0.30; Barn Swallow *Hirundo rustica*: n_males_ = 8, n_females_ = 8, W = 34, p = 0.88; Northern Wheatear *Oenanthe oenanthe*: n_males_ = 18, n_females_ = 7, W = 74, p = 0.53; Snow Bunting *Plectrophenax nivalis*: n_males_ = 8, n_females_ = 8, W = 37, p = 0.89).

### Ecological, morphological, and behavioural traits affecting seasonal differences in migration speed

In a species-level analysis, variation in the ratio between spring and autumn total speed of migration (*Q*_*speed of migration*_, expressed as the median of individual values for each species) was not found to be significantly explained by the considered ecological (breeding latitude: F_1,54_ = 0.01, P = 0.94, flight style: F_3,54_ = 0.87, P = 0.47, migration strategy: F_1,54_ = 0.11, P = 0.74, migration distance: F_1,54_ = 0.13, P = 0.72) and morphological (body mass: F_1,54_ = 1.15, P = 0.29) traits of the species in a generalized least squares regression model accounting for species’ phylogenetic relationships.

**Figure 4:**
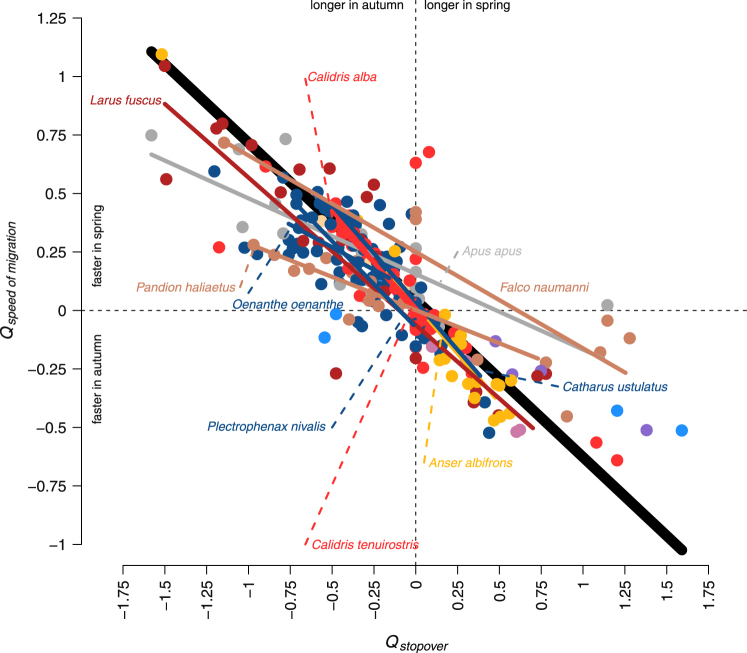
Individual ratio between spring and autumn total speed of migration (*Q*_*speed of migration*_) against individual ratio between spring and autumn total stopover duration (*Q*_*stopover*_). *Q*_*speed of migration*_ and *Q*_*stopover*_ were both estimated as the log-ratio between corresponding individual values for spring and autumn. In this phylogenetic regression with intraspecific sampling errors involved in total 257 individual tracks of 42 studies, considering 22 tracks of waterfowl (orange), 4 of bustards (purple), 5 of storks (light blue), 23 of raptors (light brown), 47 of waders (light red), 27 of gulls (dark red), 4 of owls (light purple), 17 of swifts (grey), 4 of rollers (light beige), and 104 of songbirds (dark blue). The regression line based on all species and controlling for their phylogenetic relationship is given as a thick, black line; regression lines based on single species are coloured correspondingly to their bird group and species names are shown. The negative slopes demonstrate that a shorter total stopover duration in spring resulted in a higher total speed of migration in spring.

At the individual level, variation in the ratio between spring and autumn total speed of migration (*Q*_*speed of migration*_) was modelled for 257 individuals belonging to 10 different bird groups (Fig. 4) with a phylogenetically generalized least squares model with within-species sampling error. *Q*_*speed of migration*_ was significantly negatively explained by the variation in *Q*_*stopover*_ (Likelihood ratio test: Χ^2^ = 21.4, P < 0.0001, intercept = 0.045, slope = −0.672; Fig. 4). This means that shorter total stopover duration in spring vs. autumn yielded significantly higher total speed of migration in spring than in autumn across all species. Within-species analyses, carried out for those species with at least seven tracked individuals, yielded qualitatively similar results (Table 2, Fig. 4). In a linear regression model distinguishing between within- versus between-species effects (F_2,246_ = 282, R^2^ = 0.70) but not accounting for shared ancestry, both effects were significant (within-species effect: −0.47 ± 0.026, t = −17.8, df = 243, P < 0.0001; between-species effect: −0.52 ± 0.33, t = −15.7, df = 243, P < 0.0001). Their corresponding 95% confidence intervals (95% CI) overlapped, so that the effects were not significantly different from each other (within-species effect: 95% CI −0.52–−0.42; between-species effect: 95% CI −0.59–−0.45).

**Table 2:** The effect of the ratios between the total stopover duration for spring and autumn (*Q*_*stopover*_) on the ratios between the total speed of migration for spring and autumn (*Q*_*speed of migration*_) for ten species with more than six individuals tracked. Sample size (n), estimate ± standard error (se), R^2^, and P-value are given for each linear model.

Species	n	Estimate ± se	R^2^	P
Greater White-fronted Goose *Anser albifrons*	17	−0.84 ± 0.13	0.73	<0.0001
Osprey *Pandion haliaetus*	15	−0.28 ± 0.04	0.79	<0.0001
Lesser Kestrel *Falco naumanni*	7	−0.41 ± 0.12	0.71	0.0188
Great Knot *Calidris tenuirostris*	7	−0.76 ± 0.6	0.97	<0.0001
Sanderling *Calidris alba*	13	−0.88 ± 0.09	0.92	<0.0001
Lesser Black-backed Gull *Larus fuscus*	17	−0.63 ± 0.07	0.83	<0.0001
Common Swift *Apus apus*	17	−0.23 ± 0.06	0.63	0.0002
Swainson’s Thrush *Catharus ustulatus*	37	−0.82 ± 0.06	0.86	<0.0001
Northern Wheatear *Oenanthe oenanthe*	20	−0.39 ± 0.16	0.14	0.0298
Snow Bunting *Plectrophenax nivalis*	16	−0.74 ± 0.08	0.86	<0.0001

## Discussion

The results suggest that total speed of migration was generally higher in spring than in autumn based on individual seasonal comparisons (Fig. 1), supporting former findings at the population level^1,33^. This general pattern was, however, found to be only significant in waders, gulls, swifts, and songbirds (Table 1, Fig. 3) indicating that analysing seasonal differences in total speed of migration across all species may be an oversimplification. Waterfowl and owls even travelled significantly faster in autumn than in spring^8^. The lack of a consistent pattern across the bird groups might suggest that the evolutionary benefit of travelling fast in spring may differ between groups and/or species (Table 1, Figs 1–3). Although this contrasts with the generally assumed pattern of migrating faster in spring than in autumn, such exceptions might be anticipated considering the large variation in life history of migratory birds^34^. The selected ecological and morphological traits, however, did not seem to capture the large variation in life history in the present study. This may be accounted for by the limitation of the available data, i.e., samples were not evenly distributed across the bird groups, and the considered traits and the spatiotemporal resolutions of migration might not have been sufficient to capture the differences in life history of the species involved. Regarding the proximate, behavioural mechanism, the between-individual variation in the ratio between spring and autumn total speed of migration (*Q*_*speed of migration*_) was strongly predicted by the individual ratios between spring and autumn total stopover duration (*Q*_*stopover*_) (Fig. 4).

Waterfowl, mainly represented here by Greater White-fronted Geese *Anser albifrons* (Table S1, Fig. 3), migrated faster in autumn (Fig. 2), thus contradicting the general pattern. Kölzsch *et al*.^8^ argued that migrants breeding in Arctic regions suffer from unfavourable weather^10^, food shortage, and possibly higher predation risk^35^ when arriving too early at stopovers and/or breeding areas^36^. In Bewick’s Swans *Cygnus columbianus bewickii*, total speed of migration was constrained by the speed with which the ice front retreated northwards making aquatic food sources accessible^10^. Despite the potential disadvantages of early arrival, a delayed start of spring migration is unlikely to evolve as environmental conditions en route (rather than on the wintering grounds) predict when to best arrive at the breeding areas^37–40^. Thus, starting migration early and adjusting total speed of migration to the momentarily encountered environment seems to be the most favourable strategy for arriving in time at the breeding area to maximize individual fitness. Furthermore, some species travel with a surplus of energy stores required upon arrival for clutch initiation and initial incubation (“capital breeders”)^41^. This increases energy cost of transportation which in turn might be minimized by reducing total speed of migration^6^. In the analysis on how ecological and morphological traits may affect *Q*_*speed of migration*_, latitude of the breeding area was not found to have a general effect. Probably, the mixture of species with completely different nutritional requirements hampered the detection of breeding latitude effects on *Q*_*speed of migration*_. In general, it should be emphasized that among-species variation in seasonal differences in migration speed may be related to seasonal-specific environmental constraints, such as wind conditions, ecological barriers, or ecological conditions en route, that may impede faster spring than autumn migration^8,10,35–39,42^.

The complied data set on seasonal differences in total speed of migration may suggest that a similar pattern, with higher speed in autumn, is to be found in tubenoses, bustards, and owls, whereas in storks, cuckoos, rollers, and hoopoes individuals seem to generally migrate faster in spring (Fig. 2). I would like to point out that these patterns originated from a few individuals of a low number of studies only (Fig. 3) and could potentially change with more data to come.

In waders, gulls, swifts, and songbirds, total speed of migration was significantly higher in spring in most species/populations (Table 1, Figs 2 and 3). These four bird groups represent 69% of all considered studies and 67% of all individuals (Table 1). Thus, when analysing all data combined (Fig. 1a), these significant biases strongly disguise the opposite patterns observed in the other bird groups. These biases further explain why higher migration speeds in spring were commonly assumed to well describe the generally valid seasonal speed pattern in birds^1^, even though it is not found in all groups^8–15^ (Figs 2 and 3). In contrast to the general pattern of higher migration speed in spring, as found in these four bird groups (Fig. 2), there are some studies clearly demonstrating a far higher total speed of migration in autumn (Fig. 3). In three species, Whimbrel *Numenius phaeopus*^13^, Lesser Black-backed Gull *Larus fuscus fuscus*^14^, and Blackpool Warbler *Setophaga striata*^15^, a peculiar migratory behaviour has been documented, with long non-stop flights in autumn but several shorter hops in spring^13–15^ reducing total speed of migration in spring at first glance (Fig. 3). However, the time-consuming periods when these birds fuelled for their long non-stop flights in autumn actually belong to the migration phase. If these inevitable and probably extended periods of fuelling before the first long migratory flight(s) occurred at or in the close vicinity of their breeding areas, the applied tracking techniques would not captured the actual onset of migration. Consequently, total duration of autumn migration could be significantly underestimated in these cases.

The methodological issue of correctly identifying the actual onset of migration holds true for both spring and autumn. Misidentifying the seasonal onset of migration by not capturing the pre-migratory fuelling period is probably the main reason for some extraordinarily high seasonal differences in total speed of migration and for some of the between-individual variation within a study (Fig. 3). Properly classifying when the life-history stage, migration, starts and terminates within an individual remains a major challenge because of technical limitations to detect these overlapping life-history stages^43^. Even if these difficulties were overcome, we need to be aware that we are simply comparing the observed outcome of a migratory trait, here total speed of migration. Seasonal different outcomes are then quite often interpreted as seasonally different underlying strategies. However, such a seasonal comparison does not give sufficient evidence that e.g. faster migration in spring is ultimately explained by a time minimizing strategy and slower migration in autumn by an energy minimizing strategy. For instance, birds may tend to minimize time spent for migration in autumn, but specific environmental constraints (food availability, predation risk, wind conditions) may prevent them from being faster than in spring. Therefore, an observed seasonal difference in any migratory trait does not necessarily unveil the ultimate causes underlying this change in behaviour.

The proximate, behavioural mechanism of how to adjust the ratio between spring and autumn total speed of migration (*Q*_*speed of migration*_) was mainly through variation in the ratio between spring and autumn total stopover duration (*Q*_*stopover*_) across all species (Fig. 4). The seasonal change in total stopover duration (*Q*_*stopover*_) was the main driver shaping on average 74% of the variance in seasonal difference of total speed of migration (*Q*_*speed of migration*_) in the species with more than six bird tracks (Table 2). This phenomenon was common to all bird groups suggesting that seasonal variation in total stopover duration is a general biological mechanism that explains seasonal differences in migration speed (Fig. 4)^17^, while variation in travel speed seems to be only of minor importance^1^. It seems thus surprising that migrants commonly invest in higher travel speed in spring by increasing air speed^16,44,45^ and/or prolonging flight periods per travel day^46^. However, the evolutionary benefit of this investment is not related to the absolute seasonal advancement but to the advantage of arriving before “competitors” at the breeding area^2,3^. Thus, selection favours individuals with higher travel speed under comparable conditions, because they benefit from earlier breeding area arrival relative to the others^2,3^. Investing in higher travel speed therefore seems to pay off in spring but likely less so in autumn, suggesting that short-term variation (few days) in arrival timing at the wintering ground may have smaller fitness consequences than similar variation in arrival timing at the breeding area.

## Methods

### Individual tracking data

64 studies (59 species) of 401 bird tracks provided individual estimates of total speed of migration (km/day) for consecutive seasons (Table 1, S1). Estimates of total speed of migration were either stated within the study for each species/population or calculated by dividing the individual’s total duration of migration (day) by its corresponding total migration distance (km). 42 out of the 64 studies additionally provided individual estimates (n = 257) of total stopover duration (day) for consecutive seasons. For these travel speed (km/day) was calculated as total migration distance divided by the difference of total migration duration and total stopover duration. Sex was provided for 308 (203 males, 105 females) out of the 401 individuals (Table 1). In 22 studies, at least one male and one female were tracked.

There are obvious differences in the accuracy of estimating total migration distances and identifying stopovers depending on the choice of the tracking device^47^. There was not effect of the type of the tracking device on the variation in seasonal differences in total speed of migration (ANOVA: F_2,56_ = 0.4, P = 0.68). All individual data as used in this study are provided in the supplemental data file “seasonal differences in individual total speed of migration.csv”.

### Ecological and morphological traits of the species

I classified for each bird species four ecological traits: (1) Latitude of the breeding area (°). In the analysis, I did not distinguish between the hemispheres, because only two breeding areas were located south of the equator and because the distance to the equator in general affects the more general climatic conditions of the breeding area. This approach further yielded far better residual distributions not violating model’s assumptions than considering the hemisphere of the breeding areas, details not shown. (2) Bird’s flight style: (a) continuously flapping flyers (e.g. geese, waders, gulls, terns), (b) flap-gliding flyers with irregularly long flapping and gliding phases, between which speed undulates but height does not (e.g. swifts^48^, small raptors^49^, swallows, starlings^50^), (c) bounding flyers with regular alternation of flapping and bounding (e.g. owls, cuckoos, rollers, hoopoes, songbirds excluding swallows, wood-swallows, and starlings^50^), or (d) soaring flyers (e.g. storks, large raptors^18,51^). (3) Migration strategy: either following a stop-and-go strategy (e.g. songbirds) or performing long non-stop flights lasting longer than one day (e.g. waders). Travel speed, an important ecological trait describing the migratory performance of a species, was not considered as an explanatory variable here. Its calculation and the one of total speed of migration are both based on total migration distance so that these two variables are not independent. (4) Migration distance (km): I considered the median of the spring and autumn values per species/population.

I estimated for each bird species its body mass (g). In migratory birds, this trait considerably alters in course of the year^52^ and might increase by more than 100% in relation to lean conditions during migration^26^. Hence, body mass measured at a certain date within a year does not represent species’ average value. To unbiasedly compare body mass between species and studies, I used the average value of the species and also sex, when appropriate, as given in the corresponding species account of the Handbook of the Birds of the World^53^. All traits are detailed for each species in Table S1.

### Statistics

Statistics were calculated using R 3.2.1^54^. To assess my first objective, I compared individual speed estimates of both seasons separately for the different bird groups and per species/population by using Wilcoxon signed rank tests for paired comparisons. I further ran a one-sided Wilcoxon signed rank test to assess the hypothesis that total speed of migration was higher in spring than in autumn separately for all species/populations (Table S1). To analyse these results, I applied the weighted Z-method to combine the multiple tests of the same hypothesis^55,56^. The square root of the corresponding sample size was used as the species-/population-specific weight^57^. Nine species/populations were omitted, because the corresponding P-value was 1, cf. Table S1. In general, I could not run linear models, because the critical assumption of normally distributed errors was violated regardless of how the dependent variable, i.e., total speed of migration, was transformed. Due to the low sample size in many species/populations, the median and first, third quartiles of the seasonal total speed of migration were given for each species/population to describe the corresponding distribution (Table 1).

To assess my second and third objective, I first calculated the seasonal differences in total speed of migration as:

1 $${Q}_{speedofmigration}=log10(\frac{total\,speed\,of\,migratio{n}_{spring}}{total\,speed\,of\,migratio{n}_{autumn}})$$

and similarly the seasonal ratios in total stopover duration. In some individuals total stopover duration was estimated to be zero. Because the decimal logarithm of zero is not defined, I therefore added 1 stopover day to all total stopover durations:

2 $${Q}_{stopover}=log10(\frac{total\,stopover\,duratio{n}_{spring}+1}{total\,stopover\,duratio{n}_{autumn}+1})$$

Comparative analyses including different species require to control for the effect of species’ phylogenetic relationships^58^. The phylogenetic tree of the species involved was derived from TIMETREE (http://timetree.org)^59,60^ (Fig. S1).

The variation in the seasonal differences in total speed of migration (*Q*_*speed of migration*_) between species was modelled using a generalized least squares (GLS) regression model, function “gls” of the R packages “nlme”^61^. This model allows correlated errors and unequal variances. The median seasonal difference in total speed of migration (*Q*_*speed of migration*_) was calculated for each species, but not for each population, because the phylogenetic relationship only provides a model for expected covariation on the species level. Having different populations and not species as tips in the phylogeny would involve arbitrary assumptions about the variation between populations. The variation between the species’ seasonal differences in total speed of migration (*Q*_*speed of migration*_) was related to ecological (breeding latitude, migration strategy, migration distance, flight style) and morphological (body mass) traits of each species (Table S1). Of these traits between-individual variation is pronounced in breeding latitude and body mass. In the considered species/populations, variation in breeding latitude within a species was zero in 63% of the species and below 2° in 82%, because individuals were usually originating from one single breeding area. In migratory birds, body mass dramatically changes in course of migration, so that the individual body mass had to be unified per species and if appropriate per sex (Table S1). For both numeric variables, I calculated the corresponding median per species. The three explanatory variables, breeding latitude, migration distance (log10-transformed), and body mass (log10-transformed), were tested against one another for collinearity with the “vif” function of the R package “usdm”^62^. Collinearity did not exceed 1.14; the variables were therefore treated as not collinear^63^. I accounted for phylogenetic relationships between species by including a within-group correlation structure. This correlation structure is defined by the phylogenetic tree (s. above, Fig. S1) and was here customized with the Brownian correlation structure^58^. The residual analysis of the model heavily violated model’s assumption of normally distributed residuals, details not shown. As no transformation of the numeric variables improved the residual analysis, I removed the four species (*Anser indicus, Circus pygargus, Numenius phaeopus, Tyrannus verticalis*) whose residuals were responsible for the violation. The corresponding model did not harm model’s assumption of normally distributed residuals, details not shown.

The variation in the seasonal difference of total speed of migration (*Q*_*speed of migration*_) between individuals of different species was modelled using phylogenetically generalized least squares (PGLS) with within-species sampling error^32^. For this I used the “pgls.Ives” function from the R-package “phytools”^64^, because it accounts for the phylogenetic relationship between the species and additionally for intraspecific variation in the dependent and explanatory variable, and sampling errors are allowed to be correlated. The explanatory variable was here the individual seasonal differences in total stopover duration (*Q*_*stopover*_). To the best of my knowledge, only bivariate regression models are currently able to run with “pgls.Ives”, as multivariate regressions considering sampling errors of more than one explanatory variable have not yet been implemented^64^. Therefore, the effects of ecological and morphological traits acting on the variation in the seasonal difference of total speed of migration were assessed with the GLS analysis described above. To analyse with the pgls.Ives” function whether the explanatory variable significantly affected the dependent variable, I ran a zero slope model and a variable slope model. Then I applied a likelihood ratio test to assess which model fitted better to the data^32,64,65^. Further, to evaluate the potential effect of *Q*_*stopover*_ on *Q*_*speed of migration*_ at the species level, I first selected ten species detailing more than six bird tracks for two consecutive seasons. These were *Anser albifrons*, Apus *apus*, *Calidris alba*, *C. tenuirostris*, *Catharus ustulatus*, *Falco naumanni*, *Larus fuscus*, *Oenanthe oenanthe*, *Pandion haliaetus*, and *Plectrophenax nivalis* (Table 2). I ran species-specific linear regression models with *Q*_*stopover*_ as the explanatory and *Q*_*speed of migration*_ as the independent variable. Additionally, I distinguished between within- versus between-species effects of *Q*_*stopover*_ on variation in *Q*_*speed of migration*_ following van de Pol & Wright^66^. In the corresponding linear regression model, I considered species with more than two individuals tracked and included one variable capturing the within-species variation in *Q*_*stopover*_ (differences of individual values from the species’ mean value) and another capturing the between-species variation (species’ mean value of *Q*_*stopover*_)^66^.

## Electronic supplementary material

Supplementary Information

Supplementary dataset 1
