# Supplementary material for: Proximate mechanisms affecting seasonal differences in migration speed of avian species
Source: Sci Rep. 2018 Mar 7;8:4106. doi: 10.1038/s41598-018-22421-7 (PMC5841396; doi:10.1038/s41598-018-22421-7)
Supplement: Supplementary file 1 — Supplementary Information [file 41598_2018_22421_MOESM1_ESM.doc]

Supplementary Information

**Proximate mechanisms affecting seasonal differences in migration speed of avian species**

Heiko Schmaljohann^1.2*^

^1^ Institute of Avian Research “Vogelwarte Helgoland”, An der Vogelwarte 21, 26386 Wilhelmshaven, Germany

^2^ Institute for Biology und Environmental Sciences (IBU), Carl von Ossietzky University of Oldenburg, Carl-von-Ossietzky-Straße 9-11, D-26129 Oldenburg, Germany

*Corresponding author:

[heiko.schmaljohann@uni-oldenburg.de](mailto:heiko.schmaljohann@uni-oldenburg.de)

**CONTENT**

**Table S1.** **List of studies with individual total speed of migration estimates for spring and autumn.**

**Figure S1. Relationships among bird species as considered in this study.**

**Table S1.** **List of studies with individual total speed of migration estimates for spring and autumn.** Individual sample size, study’s median of the seasonal estimates for total speed of migration (speed) and total migration distance are given for both seasons. Birds were either tracked by satellite (sat), global positioning system (gps) or light-level geolocation (llg) devices. Latitude of breeding area was given in the references. Bird’s flight style was divided into continuously flapping flyers (cf), flap-gliding flyers (fb) with irregularly long flapping and gliding phases, between which speed undulates but height does not, bounding flyers (bf) with regular alternation of flapping and bounding, or soaring flyers (s). Bird’s migration strategy was categorized as “non-stop”, i.e., performing regularly migratory flight bouts lasting longer than a single day and/or night or as “stop-go”, i.e., performing nocturnal/diurnal flights during single nights/days and resting between these migratory flights. Body mass was derived from the appropriate species’ account of the Handbook of the Birds of the World.

| Bird group | Study species | Sample size (individuals) | Median spring speed (km/day) | Median autumn speed (km/day) | Wilcoxon signed rank test of spring migration speed > autumn migration speed, P-values | Median spring migration distance (km) | Median autumn migration distance (km) | Tracking device | Latitude of breeding area (°) | Flight style | Migration strategy | Body mass (g) | References |
| --- | --- | --- | --- | --- | --- | --- | --- | --- | --- | --- | --- | --- | --- |
| Anseriformes | Bar-headed Goose  *Anser indicus* | 4 | 292 | 36 | 0.313 | 1150 | 1280 | sat | 42.2 | cf | stop-go | 1500 | 1 |
| Anseriformes | Greater White-fronted Goose  *Anser albifrons* | 17 | 47 | 83 | 0.999 | 3826 | 3264 | gps | 70 | cf | stop-go | 2350 | 2 |
| Anseriformes | Scaly-sided Merganser  *Mergus squamatus* | 1 | 113 | 80 | 0.500 | 2590 | 2230 | sat | 47 | cf | stop-go | 1300 | 3 |
| Procellariiformes | Sooty Shearwater  *Puffinus griseus* | 10 | 668 | 779 | 0.958 | 14424 | 14991 | llg | -51.2 | fg | non-stop | 814 | 4 |
| Otidiformes | Houbara Bustard  *Chlamydotis undulata* | 4 | 94 | 172 | 1 | 3922 | 4067 | sat | 39 | fg | stop-go | 1450 | 5 |
| Ciconiiformes | Black Stork  *Ciconia nigra* | 5 | 163 | 281 | 0.906 | 4882 | 4480 | sat | 48.5 | s | stop-go | 3350 | 6 |
| Ciconiiformes | Abdim’s Stork  *Ciconia abdimii* | 5 | 147 | 159 | 0.688 | 3140 | 3967 | sat | 14.0 | s | stop-go | 1300 | 7 |
| Accipitriformes | Osprey.1  *Pandion haliaetus* | 7 | 254 | 175 | 0.148 | 6341 | 6339 | sat | 59.7 | fg | stop-go | m: 1400  f: 1800 | 8 |
| Accipitriformes | Osprey.2  *Pandion haliaetus* | 8 | 153 | 141 | 0.029 | 5804 | 6123 | gps | 59 | fg | stop-go | m: 1400  f: 1800 | 9 |
| Accipitriformes | Egyptian Vulture  *Neophron percnopterus* | 6 | 191 | 248 | 0.985 | 3528 | 3202 | sat | 41 | s | stop-go | 1900 | 10 |
| Accipitriformes | Red-tailed Hawk  *Buteo jamaicensis* | 8 | 143 | 129 | 0.191 | 2236 | 2102 | sat | 43.6 | s | stop-go | 1088 | 11 |
| Accipitriformes | Oriental Honey Buzzard  *Pernis ptilorhynchus* | 1 | 122 | 184 | 1 | 10651 | 9585 | sat | 36.7 | s | stop-go | 1120 | 12 |
| Accipitriformes | Pallid Harrier  *Circus macrourus* | 3 | 218 | 73 | 0.125 | 6988 | 7691 | sat | 51 | fg | stop-go | m: 315  f: 445 | 13 |
| Accipitriformes | Montagu’s Harrier  *Circus pygargus* | 9 | 98 | 147 | 0.991 | 2883 | 2491 | sat | 41 | fg | stop-go | m: 266  f: 382 | 14 |
| Accipitriformes | Peregrine Falcon  *Falco peregrinus* | 12 | 173 | 130 | 0.017 | 5104 | 5104 | sat | 68 | fg | stop-go | 975 | 15 |
| Accipitriformes | Lesser Kestrel.1  *Falco naumanni* | 3 | 390 | 258 | 0.125 | 3132 | 3536 | sat | 38.9 | fg | stop-go | m: 131  f: 173 | 16 |
| Accipitriformes | Lesser Kestrel.2  *Falco naumanni* | 7 | 191 | 211 | 0.289 | 3360 | 3160 | llg | 43.6 | fg | stop-go | m: 131  f: 173 | 17 |
| Accipitriformes | Hobby  *Falco subbuteo* | 2 | 223 | 180 | 0.250 | 8159 | 8159 | sat | 52.8 | fg | stop-go | f: 241 | 18 |
| Charadriiformes | Grey Plover  *Pluvialis squatarola* | 3 | 67 | 37 | 0.625 | 5509 | 5611 | sat | 75 | cf | non-stop | 247 | 19 |
| Charadriiformes | Common Ringed Plover  *Charadrius hiaticula* | 5 | 230 | 199 | 0.156 | 7476 | 7315 | llg | 70.3 | cf | non-stop | 60 | 20 |
| Charadriiformes | Little Ringed Plover  *Charadrius dubius* | 3 | 173 | 130 | 0.250 | 4995 | 5531 | llg | 55.7 | cf | non-stop | 40 | 21 |
| Charadriiformes | Great Knot  *Calidris tenuirostris* | 7 | 180 | 178 | 0.277 | 9597 | 9597 | llg | 67 | cf | non-stop | 182 | 22 |
| Charadriiformes | Red Knot  *Calidris canutus* | 3 | 282 | 176 | 0.125 | 14600 | 15650 | llg | 62.5 | cf | non-stop | 153 | 23 |
| Charadriiformes | Sanderling  *Calidris alba* | 13 | 339 | 170 | 0.001 | 14170 | 14552 | llg | 73.5 | cf | non-stop | 75 | 24 |
| Charadriiformes | Temminck's Stint  *Calidris temminckii* | 1 | 108 | 190 | 1 | 5070 | 6840 | llg | 60.6 | cf | non-stop | 26 | 25 |
| Charadriiformes | Long-billed Curlew  *Numenius americanus* | 2 | 119 | 26 | 0.250 | 2910 | 3312 | sat | 50 | cf | non-stop | m: 619 | 26 |
| Charadriiformes | Whimbrel  *Numenius phaeopus* | 4 | 463 | 1060 | 1 | 5460 | 5298 | llg | 63.8 | cf | non-stop | m: 409  f: 458 | 27 |
| Charadriiformes | Great Snipe  *Gallinago media* | 3 | 329 | 238 | 0.625 | 7529 | 7822 | llg | 63 | cf | non-stop | 200 | 28 |
| Charadriiformes | Red-necked Phalarope  *Phalaropus lobatus* | 3 | 159 | 197 | 0.625 | 6908 | 5709 | llg | 95.9 | cf | non-stop | 34 | 29 |
| Charadriiformes | Pallas's Gull  *Larus ichthyaetus* | 6 | 76 | 23 | 0.016 | 2695 | 1975 | sat | 36.9 | cf | non-stop | 1450 | 30 |
| Charadriiformes | Lesser Black-backed Gull.1  *Larus fuscus* | 12 | 96 | 30 | 0.005 | 2046 | 2098 | gps | 53.2 | cf | stop-go | 875 | 31 |
| Charadriiformes | Lesser Black-backed Gull.2  *Larus fuscus fuscus* | 5 | 170 | 379 | 1 | 7158 | 7158 | llg | 65.3 | cf | non-stop | 875 | 32 |
| Charadriiformes | Relict Gull  *Larus (Ichthyaetus) relictus* | 13 | 202 | 60 | 0.188 | 690 | 890 | sat | 38.2 | cf | stop-go | 523 | 33 |
| Charadriiformes | Common Tern  *Sterna hirundo* | 2 | 320 | 300 | 0.500 | 12450 | 12450 | llg | 41.7 | cf | stop-go | 122 | 34 |
| Charadriiformes | Black Tern  *Chlidonias niger* | 3 | 339 | 200 | 0.125 | 6750 | 6500 | llg | 52 | cf | stop-go | 67 | 35 |
| Strigiformes | Flammulated Owl  *Otus (Psiloscops) flammeolus* | 5 | 59 | 165 | 1 | 2070 | 2070 | llg | 39.1 | bf | stop-go | 54 | 36 |
| Apodiformes | Common Swift.1  *Apus apus* | 6 | 322 | 146 | 0.016 | 9466 | 9338 | llg | 55.5 | fg | non-stop | 44 | 37 |
| Apodiformes | Common Swift.2  *Apus apus* | 11 | 555 | 275 | 0.001 | 8800 | 8800 | llg | 51 | fg | non-stop | 44 | 38 |
| Cuculiformes | Eurasian Ccuckoo  *Cuculus canorus* | 3 | 85 | 62 | 0.125 | 9100 | 7559 | sat | 54 | bf | stop-go | 115 | 39 |
| Coraciiformes | European Roller  *Coracias garrulus* | 4 | 152 | 102 | 0.063 | 9298 | 9062 | llg | 37 | fg | stop-go | m: 194  f: 142 | 40 |
| Bucerotiformes | Hoopoe  *Upupa epops* | 2 | 142 | 100 | 0.250 | - | - | llg | 46 | bf | stop-go | 68 | 41 |
| Passeriformes | Purple Martin  *Progne subis* | 2 | 429 | 154 | 0.250 | 7550 | 6750 | llg | 41.8 | fg | stop-go | 56 | 42 |
| Passeriformes | Barn Swallow  *Hirundo rustica* | 16 | 263 | 105 | 0.0004 | 7730 | 7963 | llg | 47.7 | fg | stop-go | 20 | 43 |
| Passeriformes | Western Kingbird  *Tyrannus verticalis* | 1 | 230 | 251 | 1 | 2531 | 1507 | llg | 34.4 | bf | stop-go | 38 | 44 |
| Passeriformes | Eastern Kingbird  *Tyrannus tyrannus* | 6 | 252 | 127 | 0.016 | 5174 | 6809 | llg | 40.5 | bf | stop-go | 41 | 45 |
| Passeriformes | Fork-tailed Flycatcher  *Tyrannus savana* | 6 | 118 | 68 | 0.047 | 3190 | 3190 | llg | -22.3 | bf | stop-go | 30 | 45 |
| Passeriformes | Scissor-tailed Flycatcher  *Tyrannus forficatus* | 1 | 419 | 252 | 0.500 | 2515 | 2515 | llg | 34.4 | bf | stop-go | 40 | 46 |
| Passeriformes | Swainson's Thrush.1  *Catharus ustulatus* | 8 | 106 | 56 | 0.004 | 5048 | 4744 | llg | 49.3 | bf | stop-go | 35 | 45 |
| Passeriformes | Swainson's Thrush.2  *Catharus ustulatus* | 29 | 131 | 73 | 0.001 | 6309 | 5626 | llg | 50 | bf | stop-go | 35 | 47 |
| Passeriformes | Veery  *Catharus fuscescens* | 3 | 271 | 89 | 0.125 | 6885 | 7041 | llg | 39.4 | bf | stop-go | 34 | 48 |
| Passeriformes | Great Reed Warbler  *Acrocephalus arundinaceus* | 6 | 218 | 151 | 0.016 | 7004 | 6121 | llg | 59.2 | bf | stop-go | 27 | 49 |
| Passeriformes | Northern Wheatear.1  *Oenanthe oenanthe* | 5 | 158 | 85 | 0.063 | 4380 | 4025 | llg | 49.5 | bf | stop-go | 26 | 50 |
| Passeriformes | Northern Wheatear.2  *Oenanthe oenanthe* | 12 | 163 | 86 | 0.0002 | 5299 | 5186 | llg | 59.8 | bf | stop-go | 26 | 51 |
| Passeriformes | Northern Wheatear.3  *Oenanthe oenanthe* | 8 | 190 | 157 | 0.027 | 7742 | 15252 | llg | 66 | bf | stop-go | 26 | 52 |
| Passeriformes | Red-spotted Bluethroat  *Luscinia svecica* | 1 | 209 | 77 | 0.50 | 5636 | 5636 | llg | 50.7 | bf | stop-go | 18 | 53 |
| Passeriformes | Red-backed Shrike  *Lanius collurio* | 6 | 189 | 100 | 0.016 | 11732 | 10050 | llg | 56 | bf | stop-go | 28 | 54 |
| Passeriformes | Red-eyed Vireo  *Vireo olivaceus* | 1 | 130 | 141 | 1 | 6631 | 6631 | llg | 41.8 | bf | stop-go | 19 | 55 |
| Passeriformes | Semicollared Flycatcher  *Ficedula semitorquata* | 10 | 106 | 67 | 0.003 | 5350 | 5782 | llg | 42.9 | bf | stop-go | 13 | 56 |
| Passeriformes | Pied Flycatcher  *Ficedula hypoleuca* | 8 | 323 | 135 | 0.004 | 5018 | 5018 | llg | 52.8 | bf | stop-go | 16 | 57 |
| Passeriformes | Tawny Pipit  *Anthus campestris* | 4 | 104 | 86 | 0.188 | 4475 | 4100 | llg | 50 | bf | stop-go | 25 | 58 |
| Passeriformes | Blackpoll Warbler  *Dendroica (Setophaga) striata* | 3 | 195 | 325 | 1 | 3900 | 3900 | llg | 44 | bf | non-stop | 15 | 59 |
| Passeriformes | Chestnut-cheeked Starling  *Sturnus (Agropsar) philippensis* | 3 | 164 | 191 | 0.875 | 4800 | 4800 | llg | 38 | fg | stop-go | 50 | 60 |
| Passeriformes | Common Rosefinch  *Carpodacus erythrinus* | 3 | 207 | 83 | 0.125 | 6390 | 6204 | llg | 57 | bf | stop-go | 26 | 61 |
| Passeriformes | Linnet  *Carduelis (Linaria) cannabina* | 5 | 20 | 29 | 0.688 | 1383 | 1383 | llg | 54 | bf | stop-go | 21 | 62 |
| Passeriformes | Ortolan Bunting  *Emberiza hortulana* | 6 | 146 | 110 | 0.031 | 6101 | 6101 | llg | 63 | bf | stop-go | 23 | 63 |
| Passeriformes | Snow Bunting *Plectrophenax nivalis* | 17 | 100 | 81 | 0.007 | 2100 | 2700 | llg | 64 | bf | stop-go | 67 | 64 |

**Figure S1. Relationships among bird species as considered in this study.** Data were downloaded from [www.timetree.org](http://www.timetree.org/). The Newick code including the phylogenetic data is displayed below.


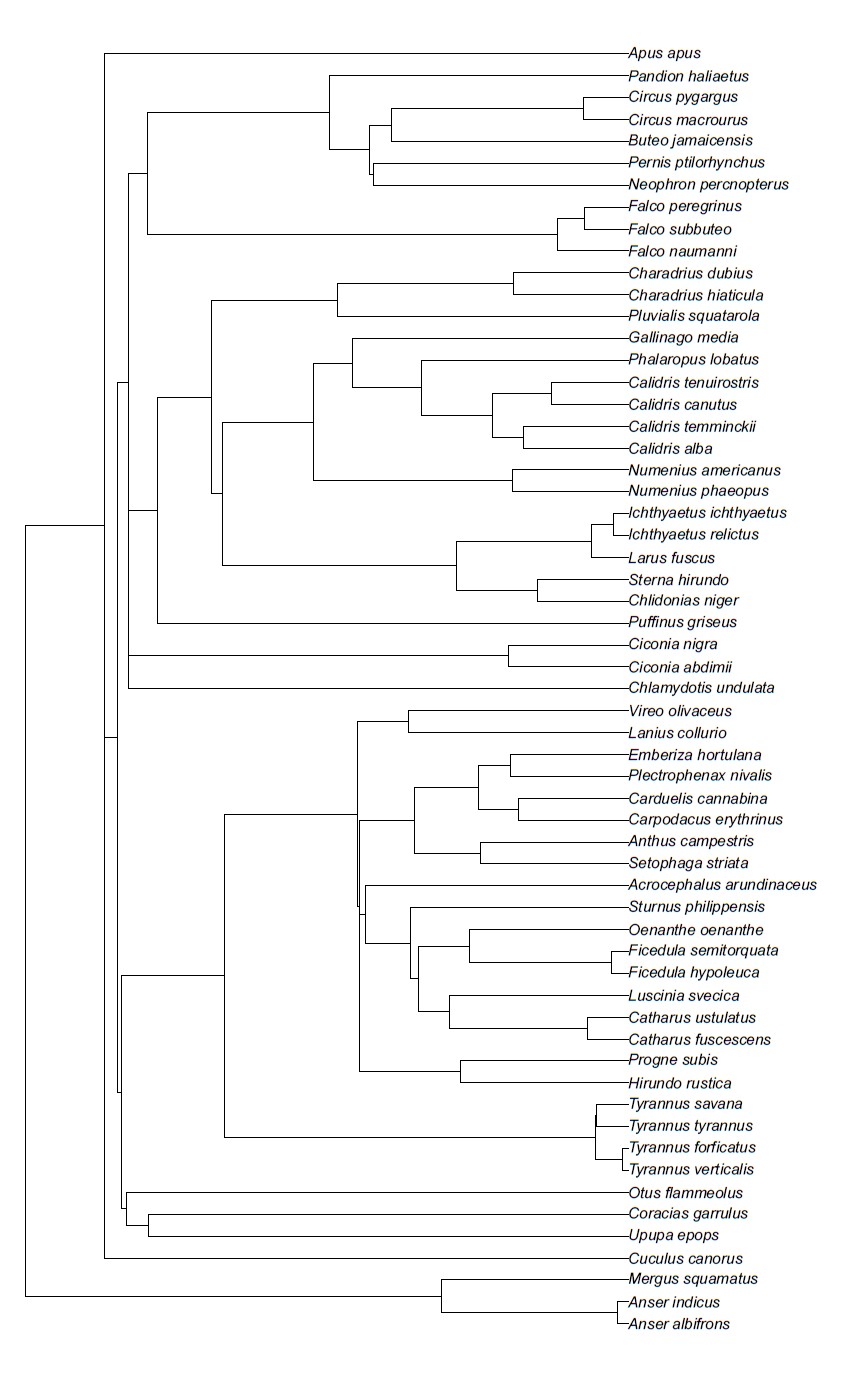


Newick file: (((Anser_albifrons:1.77647200,Anser_indicus:1.77647200)'14':28.55229444,Mergus_squamatus:30.32876644)'13':67.71410286,((Cuculus_canorus:85.20000000,((((Upupa_epops:78.01644867,Coracias_garrulus:78.01644867)'11':3.58355133,Otus_flammeolus:81.60000000)'10':0.85491770,(((Tyrannus_verticalis:0.87126700,Tyrannus_forficatus:0.87126700)'19':4.49873300,(Tyrannus_tyrannus:5.21485000,Tyrannus_savana:5.21485000)'9':0.15515000)'22':60.32121089,((((Hirundo_rustica:27.33644000,Progne_subis:27.33644000)'8':16.36356000,(((((Catharus_fuscescens:6.69128500,Catharus_ustulatus:6.69128500)'6':22.32099875,Luscinia_svecica:29.01228375)'30':5.18771625,((Ficedula_hypoleuca:2.72288567,Ficedula_semitorquata:2.72288567)'29':23.17544933,Oenanthe_oenanthe:25.89833500)'27':8.30166500)'35':1.24880857,Sturnus_philippensis:35.44880857)'43':7.28981874,Acrocephalus_arundinaceus:42.73862731)'42':0.96137269)'40':0.00000000,((Setophaga_striata:24.06782500,Anthus_campestris:24.06782500)'48':10.73217500,((Carpodacus_erythrinus:17.90000000,Carduelis_cannabina:17.90000000)'51':6.37687333,(Plectrophenax_nivalis:19.20000000,Emberiza_hortulana:19.20000000)'47':5.07687333)'39':10.52312667)'56':8.90000000)'55':0.30000000,(Lanius_collurio:35.80000000,Vireo_olivaceus:35.80000000)'61':8.20000000)'60':21.69121089)'54':16.76370681)'38':0.65432695,(((Chlamydotis_undulata:81.30000000,(Ciconia_abdimii:19.50000000,Ciconia_nigra:19.50000000)'34':61.80000000)'26':0.00000000,(Puffinus_griseus:76.59134568,((((Chlidonias_niger:14.67847333,Sterna_hirundo:14.67847333)'66':13.26795827,(Larus_fuscus:5.93000000,(Ichthyaetus_relictus:2.37546900,Ichthyaetus_ichthyaetus:2.37546900)'75':3.55453100)'80':22.01643160)'78':38.08830017,((Numenius_phaeopus:18.85661000,Numenius_americanus:18.85661000)'74':32.28975667,((((Calidris_alba:16.98141000,Calidris_temminckii:16.98141000)'83':5.11859000,(Calidris_canutus:12.43388000,Calidris_tenuirostris:12.43388000)'73':9.66612000)'88':11.54641717,Phalaropus_lobatus:33.64641717)'86':11.18481083,Gallinago_media:44.83122800)'72':6.31513867)'93':14.88836510)'92':1.80620966,(Pluvialis_squatarola:47.30658750,(Charadrius_hiaticula:18.70734333,Charadrius_dubius:18.70734333)'91':28.59924417)'71':20.53435393)'69':8.75040425)'65':4.70865432)'25':0.00000000,((Falco_naumanni:11.42913250,(Falco_subbuteo:7.12781500,Falco_peregrinus:7.12781500)'5':4.30131750)'102':66.84231836,(((Neophron_percnopterus:41.46281000,Pernis_ptilorhynchus:41.46281000)'100':0.68730200,(Buteo_jamaicensis:38.56443400,(Circus_macrourus:7.19275000,Circus_pygargus:7.19275000)'107':31.37168400)'111':3.58567800)'110':6.38300438,Pandion_haliaetus:48.53311638)'106':29.73833447)'129':3.02854914)'128':1.80924465)'133':2.09075535)'127':0.00000000,Apus_apus:85.20000000)'136':12.84286929);

**References**

1 Köppen, U., Yakovlev, A. P., Barth, R., Kaatz, M. & Berthold, P. Seasonal migrations of four individual bar-headed geese *Anser indicus* from Kyrgyzstan followed by satellite telemetry. *J. Ornithol.* **151**, 703-712 (2010).

2 Kölzsch, A. *et al.* Towards a new understanding of migration timing: slower spring than autumn migration in geese reflects different decision rules for stopover use and departure. *Oikos* **125**, 1496-1507 (2016).

3 Dong-Ping, L., Cheng-Quan, L., Guo-Gang, Z., Jun, L. & Gang, C. Satellite tracking of Scaly-sided Merganser (*Mergus squamatus*) breeding in Lesser Xingan Mountains, China. *Waterbirds* **37**, 432-438 (2014).

4 Hedd, A., Montevecchi, W. A., Otley, H., Phillips, R. A. & Fifield, D. A. Trans-equatorial migration and habitat use by sooty shearwaters *Puffinus griseus* from the South Atlantic during the nonbreeding season. *Mar. Ecol. Prog. Ser.* **449**, 277-290 (2012).

5 Judas, J. *et al.* Migration and range use of Asian Houbara Bustard *Chlamydotis macqueenii* breeding in the Gobi Desert, China, revealed by satellite tracking. *Ibis* **148**, 343-351 (2006).

6 Chevallier, D. *et al.* Influence of weather conditions on the flight of migrating black storks. *Proc. R. Soc. Lond. B* **277**, 2755-2764 (2010).

7 Jensen, F. P., Falk, K. & B.S., P. Migration routes and staging areas of Abdim's Storks Ciconia abdimii identified by satellite telemetry. *Ostrich* **77**, 210-219 (2006).

8 Alerstam, T., Hake, M. & Kjellen, N. Temporal and spatial patterns of repeated migratory journeys by ospreys. *Anim. Behav.* **71**, 555-566 (2006).

9 Väli, Ü. & Sellis, U. Migration patterns of the Osprey *Pandion haliaetus* on the Eastern European–East African flyway. *Ostrich* **87**, 23-28 (2015).

10 López-López, P., García-Ripollés, C. & Urios, V. Individual repeatability in timing and spatial flexibility of migration routes of trans-Saharan migratory raptors. *Curr. Zool.* **60**, 642-652 (2014).

11 Craighead, D., Crandall, R. H., Smith, R. N. & Cain, S. L. Migration of red-tailed hawks (*Buteo jamaicensis*) from northwest Wyoming. *Wilson J. Ornithol.* **128**, 150-158 (2016).

12 Higuchi, H. *et al.* Migration of Honey-buzzards *Pernis apivorus* based on satellite tracking. *Ornithol. Sci.* **4**, 109-115 (2005).

13 Terraube, J. *et al.* Broad wintering range and intercontinental migratory divide within a core population of the near-threatened pallid harrier. *Divers. Distrib.* **18**, 401-409 (2011).

14 Limiñana, R., Soutullo, A., Urios, V. & Reig-Ferrer, A. Migration and wintering areas of adult Montagu’s Harriers (*Circus pygargus*) breeding in Spain. *J. Ornithol.* **153**, 85-93 (2012).

15 McGrady, M. J., Maechtle, T. L., Varags, J. J., Seegar, W. S. & Peña, M. C. P. Migration and ranging of Peregrine Falcons wintering on the Gulf of Mexico Coast. *Condor* **104**, 39-48 (2002).

16 Limiñana, R., Romero, M., Mellone, U. & Urios, V. Mapping the migratory routes and wintering areas of Lesser Kestrels *Falco naumanni*: new insights from satellite telemetry. *Ibis* **154**, 389-399 (2012).

17 Pilard, P., Bourgeois, M. & Sylla, D. Localisation des quartiers d´hivernage et phénologie des migrations prénuptiale et postnuptiale chez la population Française du faucon crécerellette *Falco naumanni* á l'aide de géolocateurs. *Alauda* **85**, 1-28 (2017).

18 Meyburg, B.-U., Howey, P. W., Meyburg, C. & Fiuczynski, K. D. Two complete migration cycles of an adult Hobby tracked by satellite. *British Birds* **104**, 2-15 (2011).

19 Exo, K.-M. Migration of Grey Plovers *Pluvialis squatarola*. (in prep).

20 Lislevand, T., Briedis, M., Heggøy, O. & Hahn, S. Seasonal migration strategies of Common Ringed Plovers *Charadrius hiaticula*. *Ibis* **159**, 225-229 (2017).

21 Hedenström, A., Klaassen, R. H. G. & Åkesson, S. Migration of the Little Ringed Plover *Charadrius dubius* breeding in South Sweden tracked by geolocators. *Bird Study* **60**, 466-474 (2013).

22 Lisovski, S., Gosbell, K., Hassell, C. & Minton, C. Tracking the full annual-cycle of Great Knot (*Calidris tenuirostris*), a long-distant migratory shorebird of the East-Asian Australasian Flyway. *Wader Study* **123**, 177-189 (2017).

23 Tomkovich, P. S., Porter, R. R., KLoktionov, E. Y. & Niles, L. J. Pathways and staging areas of Red Knots *Calidris canutus rogersi* breeding in southern Chukotka, Far Eastern Russia. *Wader Study* **120**, 181-193 (2013).

24 Lisovski, S. *et al.* Movement patterns of Sanderling (*Calidris alba*) along the East Asian Australasian Flyway and a comparison of methods to identify crucial areas for conservation. *Emu* **116**, 168-177 (2016).

25 Lislevand, T. & Hahn, S. Skipping-type migration in a small Arctic wader, the Temminck's stint Calidris temminckii. J. Avian Biol. **46**, 419-424 (2015).

26 Olalla-Kerstupp, A., Ruiz-Aymá, G., González-Rojas, J. I. & Guzmán-Velasco, A. High fidelity to wintering, stop-over and breeding sites shown by a Long-billed Curlew *Numenius americanus* tracked with satellite telemetry on migratory flights across North America. *Bird Study* **62**, 556-560 (2015).

27 Alves, J. A., Dias, M. P., Méndez, V., Katrínardóttir, B. & Gunnarsson, T. G. Very rapid long-distance sea crossing by a migratory bird. *Sci. Rep.* **6**, 38154 (2016).

28 Klaassen, R. H. G., Alerstam, T., Carlsson, P., Fox, J. W. & Lindström, Å. Great flights by great snipes: long and fast non-stop migration over benign habitats. *Biol. Lett.* **7**, 833-835 (2011).

29 van Bemmelen, R. S. A., Hungar, J., Tulp, I. & Klaassen, R. H. G. First geolocator tracks of Swedish red-necked phalaropes reveal the Scandinavia-Arabian Sea connection. *J. Avian Biol.* **46**, 1-9 (2015).

30 Guo-Gang, Z. *et al.* Migration routes and stopover sites of Pallas’s Gulls *Larus ichthyaetus* breeding at Qinghai Lake, China, determined by satellite tracking. *Forktail* **30**, 104-108 (2014).

31 Klaassen, R. H. G., Ens, B. J., Shamoun-Baranes, J., Exo, K.-M. & Bairlein, F. Migration strategy of a flight generalist, the Lesser Black-backed Gull *Larus fuscus*. *Behav. Ecol.* **23**, 58-68 (2012).

32 Bustnes, J. O., Moe, B., Helberg, M. & Phillips, R. A. Rapid long-distance migration in Norwegian Lesser Black-backed Gulls *Larus fuscus fuscus* along their eastern flyway. *Ibis* **155**, 402-406 (2013).

33 Liu, D. *et al.* Seasonal dispersal and longitudinal migration in the Relict Gull *Larus relictus* across the Inner-Mongolian Plateau. *PeerJ* **5**, e3380 (2017).

34 Nisbet, I. C. T., Mostello, C. S., Veit, R. R., Fox, J. W. & Afanasyev, V. Migrations and winter quarters of five Common Terns tracked using geolocators. *Waterbirds* **34**, 32-39 (2011).

35 van der Winden, J., Fijn, R. C., van Horssen, P. W., Gerritsen-Davidse, D. & Piersma, T. Idiosyncratic migrations of Black Terns (*Chlidonias niger*): Diversity in routes and stopovers. *Waterbirds* **37**, 162-174 (2014).

36 Linkhart, B. D., Fox, J. W. & Yanco, S. W. Migration timing and routes, and wintering areas of Flammulated Owls. *J. Field Ornithol.* **87**, 42-54 (2016).

37 Åkesson, S., Klaassen, R. H. G., Holmgren, J., Fox, J. W. & Hedenström, A. Migration routes and strategies in a highly aerial migrant, the Common Swift *Apus apus*, revealed by light-level geolocators. *PlosOne* **7**, e41195 (2012).

38 Wellbrock, A. H. J., Bauch, C., Rozman, J. & Witte, K. “Same procedure as last year?” – Repeatedly tracked swifts show individual consistency in migration pattern in successive years. *J. Avian Biol.* 48, 897-903 (2017).

39 Willemoes, M. *et al.* Narrow-front loop migration in a population of the Common Cuckoo *Cuculus canorus*, as revealed by satellite telemetry. *PLoSOne* **9**, e83515 (2014).

40 Rodríguez-Ruiz, J. *et al.* Disentangling migratory routes and wintering grounds of Iberian near-threatened European Rollers *Coracias garrulus*. *PLoSOne* **9**, e115614 (2014).

41 Bächler, E. *et al.* Year-round tracking of small trans-Saharan migrants using light-level geolocators. *PlosOne* **3**, e9566 (2010).

42 Stutchbury, B. J. M. *et al.* Tracking long-distance songbird migration by using geolocators. *Science* **323**, 896 (2009).

43 Hobson, K. A. *et al.* A continent-wide migratory divide in North American breeding barn swallows (*Hirundo rustica*). *PLoSOne* **70**, e0129340 (2015).

44 Jahn, A. E. *et al.* Migration timing and wintering areas of three species of flycatchers (*Tyrannus*) breeding in the Great Plains of North America. *Auk* **130**, 247-257 (2013).

45 Jahn, A. E. *et al.* Intra-tropical migration and wintering areas of Fork-tailed Flycatchers (*Tyrannus savana*) breeding in São Paulo, Brazil. *Revista Brasileira de Ornitologia* **24**, 116-121 (2016).

46 Delmore, K. E., Fox, J. W. & Irwin, D. E. Dramatic intraspecific differences in migratory routes, stopover sites and wintering areas, revealed using light-level geolocation. *Proc. R. Soc. Lond. B* **279**, 4582-4589 (2012).

47 Delmore, K. E. & Irwin, D. E. Hybrid songbirds employ intermediate routes in a migratory divide. *Ecol. Lett.* **17**, 1211-1218 (2014).

48 Heckscher, C. M., Taylor, S. M., Fox, J. W. & Afanasyev, V. Veery (*Catharus fuscescens*) wintering locations, migratory connectivity, and a revision of its winter range using geolocator technology. *Auk* **128**, 531-542 (2011).

49 Lemke, H. W. *et al.* Annual cycle and migration strategies of a trans-Saharan migratory songbird: A geolocator study in the great reed warbler. *PLoSOne* **8**, e79209 (2013).

50 Schmaljohann, H., Buchmann, M., Fox, J. W. & Bairlein, F. Tracking migration routes and the annual cycle of a trans-Sahara songbird migrant. *Behav. Ecol. Sociobiol.* **66**, 915-922, (2012).

51 Arlt, D., Olsson, P., Fox, J. W., Low, M. & Pärt, T. Prolonged stopover duration characterises migration strategy and constraints of a long-distance migrant songbird. *Anim. Mig.* **2**, 47-62 (2015).

52 Schmaljohann, H., Lisovski, S. & Bairlein, F. Flexible reaction norms to environmental variables along the migration route and the significance of stopover duration for total speed of migration in a songbird migrant. *Front. Zool.* **14:17** (2017).

53 Lislevand, T. *et al.* Red-spotted Bluethroats *Luscinia s. svecica* migrate along the Indo-European flyway: a geolocator study. *Bird Study* **62**, 508-515 (2015).

54 Tøttrup, A. P. *et al.* The annual cycle of a trans-equatorial Eurasian-African passerine migrant: different spatio-temporal strategies for autumn and spring migration. *Proc. R. Soc. Lond. B* **279**, 1008-1016 (2012).

55 Callo, P. A., Morton, E. S. & Stutchbury, B. J. M. Prolonged spring migration in the red-eyed vireo (*Vireo olivaceus*). *Auk* **130**, 240-246 (2013).

56 Briedis, M. *et al.* Year-round spatiotemporal distribution of the enigmatic Semi-collared Flycatcher *Ficedula semitorquata*. *J. Ornithol.* **157**, 895-900 (2016).

57 Ouwehand, J. *et al.* Light-level geolocators reveal migratory connectivity in European populations of pied flycatchers *Ficedula hypoleuca*. *J. Avian Biol.* **47**, 69-83 (2016).

58 Briedis, M., Beran, V., Hahn, S. & Adamík, P. Annual cycle and migration strategies of a habitat specialist, the Tawny Pipit *Anthus campestris*, revealed by geolocators. *J. Ornithol.* **157**, 619-626 (2016).

59 DeLuca, W. V. *et al.* Transoceanic migration by a 12 g songbird. *Biol. Lett.* **11**, 20141045 (2015).

60 Koike, S., Hijikata, N. & Higuchi, H. Migration and wintering of Chestnut-cheeked Starlings *Agropsar philippensis*. *Ornithol. Sci.* **15**, 63-74 (2016).

61 Stach, R., Kullberg, C., Jakobsson, S., Ström, K. & Fransson, T. Migration routes and timing in a bird wintering in South Asia, the Common Rosefinch *Carpodacus erythrinus*. *J. Ornithol.* **157**, 671-679 (2016).

62 Rösseler, D., Schmaljohann, H. & Bairlein, F. Timing of migration, routes and wintering grounds of a short-distance diurnal migrant revealed by geolocation: A case study of Linnets *Carduelis cannabina*. *J. Ornithol.* **158**, 875-880 (2017).

63 Selstam, G., Sondell, J. & Olsson, P. Wintering area and migration routes for Ortolan Buntings *Emberiza hortulana* from Sweden determined with light-geologgers. *Ornis Svecica* **25**, 3-14 (2015).

64 McKinnon, E. A., MacDonald, C. M., Gilchrist, H. G. & Love, O. P. Spring and fall migration phenology of an Arctic-breeding passerine. *J. Ornithol.* **157**, 681-693 (2016).
